# Two novel truncating variants in *UBAP1* are responsible for hereditary spastic paraplegia

**DOI:** 10.1371/journal.pone.0253871

**Published:** 2021-06-30

**Authors:** Xinchao Bian, Guangying Cheng, Xinbo Sun, Hongkun Liu, Xiangmao Zhang, Yu Han, Bo Li, Ning Li

**Affiliations:** 1 Department of Neurosurgery, Zibo Central Hospital, Shandong University, Zibo, China; 2 Department of Gynecology, Zibo Central Hospital, Shandong University, Zibo, China; 3 Department of Integrated Traditional Chinese and Western Medicine Orthopedics, Zibo Central Hospital, Shandong University, Zibo, China; Oslo Universitetssykehus, NORWAY

## Abstract

Hereditary spastic paraplegias (HSPs) are a group of rare neurodegenerative disorders. HSPs are complex disorders and are clinically and genetically heterogeneous. To date, more than 80 genes or genetic loci have been reported to be responsible for HSPs in a Mendelian-dependent manner. Most recently, ubiquitin-associated protein 1 (UBAP1) has been recognized to be involved in HSP. Here, we identified novel protein truncating variants in two families with pure form of HSP. A novel deletion (c.468_469delTG) in the *UBAP1* gene was found in the first family, whereas a nonsense variant (c.512T>G) was ascertained in the second family. The variants were confirmed in all patients but were not detected in unaffected family members. The mutations resulted in truncated proteins of UBAP1. The variants did not result in different subcellular localizations in neuro-2a cells. However, each of the two variants impaired neurite outgrowth. Taken together, our findings expand the pathogenic spectrum of *UBAP1* variants in HSP.

## Introduction

Hereditary spastic paraplegias (HSPs) are a group of rare neurodegenerative disorders that are characterized by progressive weakness and spasticity of the lower limbs [1]. The estimated prevalence of HSPs is 1.3–9.6 per 100,000 individuals [2]. HSPs impair the corticospinal tract and are both clinically and genetically heterogeneous [3]. Clinically, although the clinical presentation of HSPs encompasses a wide spectrum of phenotypes, they can be classified into pure forms and complicated forms. In pure forms of HSP, progressive lower-limb spasticity and weakness are the main clinical features. In complex forms of HSP, neurological or other features are also present, such as epilepsy, ataxia, and optic neuropathy [4, 5]. Genetically, HSPs consist of different inherited patterns and types. Thus far, more than 80 genes or genetic loci have been reported to be responsible for HSPs in a Mendelian-dependent manner [6]. However, no genetic diagnoses exist for approximately 40% of HSP patients even after whole-exome sequencing. In addition, many of the genes responsible for HSPs have only been reported in sporadic families [7].

In 2019, *ubiquitin-associated protein 1* (*UBAP1*) was recognized as a pathogenic gene of HSPs [2]. *UBAP1* (Genbank NM_016525), located on chromosome 9p13, is composed of 16 exons spanning 73.5 kb. It encodes UBAP1, a 502-amino-acid-residue peptide. To date, there have been 15 pathogenic variants in *UBAP1* responsible for spastic paraplegia in five separate reports [2, 8–12]. UBAP1, together with TSG101, VPS28, and VPS37A, comprise the mammalian endosomal-sorting complex required for transport I (ESCRT-I) [13]. UBAP1 is involved in endosomal dynamics in neurons [10]. Disruption of UBAP1 dysregulates early endosomal processing and ubiquitinated protein sorting [8]. However, the functional characterization of UBAP1 in HSP requires further investigation.

In this report, we screened the *UBAP1* gene in two recruited HSP families via direct sequencing. We identified two novel truncating mutations in *UBAP1* as the genetic causes of HSP in two Chinese families. We found that the two novel mutated transcripts produced truncated UBAP1. These data expand the spectrum of known causal mutations of *UBAP1*. We subsequently performed bioinformatic analysis and functional studies in neuro-2a cells. Our biochemical and cell-biology evidence revealed that UBAP1 mutants inhibited the formation and extension of neurites. To our knowledge, this is the first functional characterization of *UBAP1* revealing an influence on neurites.

## Materials and methods

### Subject recruitment and clinical evaluations

**Fig 1:**
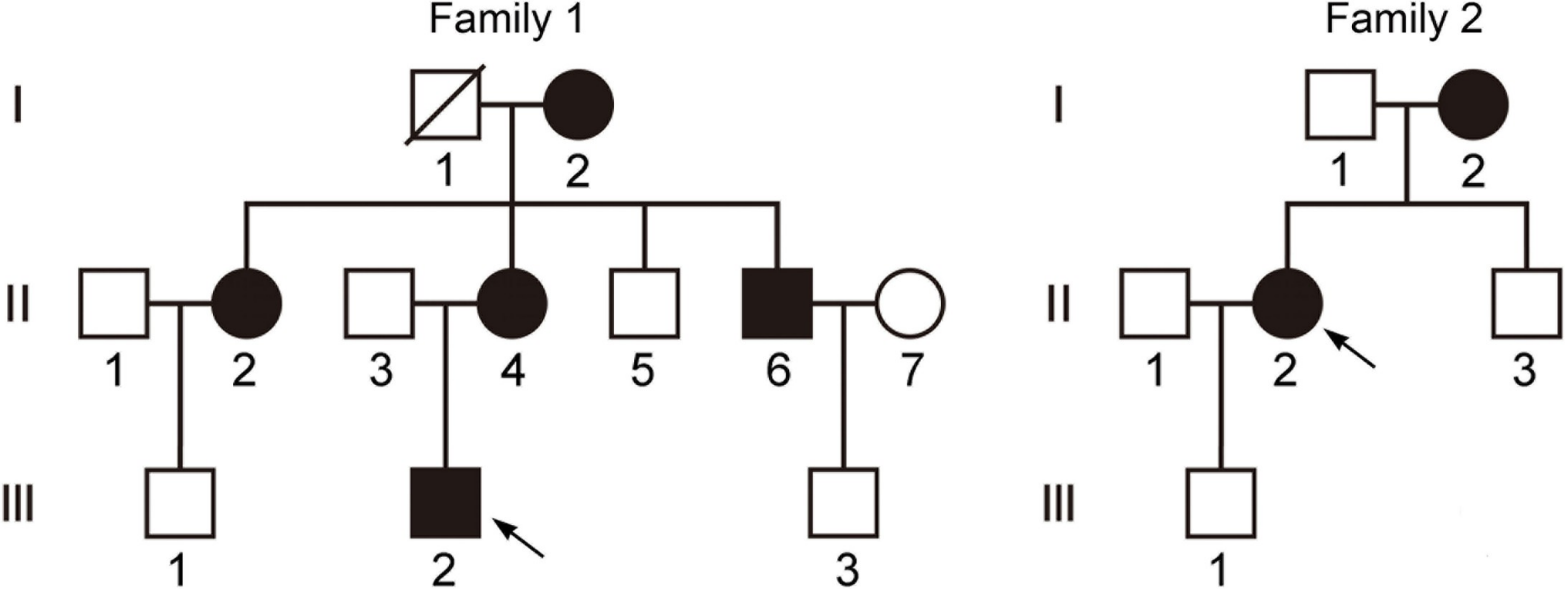
Pedigrees of family 1 and family 2 with mutations in the *UBAP1* gene. Squares and circles indicate males and females, respectively. Black symbols represent members with an HSP phenotype and empty symbols represent unaffected individuals. Arrows indicate the probands.

Two Han Chinese families with HSPs were recruited and evaluated by Zibo Central Hospital. In total, 17 family members participated in the present study, including seven affected individuals and 10 unaffected individuals (Fig 1). All participants were clinically evaluated based on their medical histories and examinations. A total of 132 unrelated Chinese individuals, without a family history of HSPs, were recruited as controls. All patients fulfilled the Harding’s classification for HSP and showed a pure form of juvenile onset (Table 1). Peripheral blood samples were collected for analysis of DNA and protein of *UBAP1*. Informed written consent was obtained from each participant following the Declaration of Helsinki. All experimental procedures were approved by the Ethics Committee of Zibo Central Hospital.

**Table 1:** Clinical features in HSP patients of the two families.

Patients	Family 1					Family 2	
	I-2	II-2	II-4	II-6	III-2	I-2	II-2
**Sex**	F	F	F	M	M	F	F
**Onset age**	NA	17	13	13	10	22	9
**Duration**	NA	24	25	19	1	29	17
**Disability score**	3	2	2	2	1	3	2
**LL hypertonia**	+	+	+	+	-	+	+
**LL hyperreflexia**	+	+	+	+	+	+	+
**Babinski sign**	+	+	+	+	-	+	+
**Muscle atrophy**	-	-	-	-	-	-	-
**Ankle clonus**	+	+	+	+	-	+	+
**Pes cavus**	-	-	-	-	-	-	-
**Cognitive impairment**	-	-	-	-	-	-	-
**Ataxia**	-	-	-	-	-	-	-
**Dysarthria**	-	-	-	-	-	-	-
**Brain MRI**	NA	NA	Normal	NA	Normal	Normal	Normal
**Electromyography**	NA	Normal	Normal	Normal	Normal	Normal	Normal
**Serum creatine kinase, IU/l**	NA	106	85.0	52.3	74.2	95.1	112

F = female; LL = lower limbs; M = male; NA, not available +, present; -, absent.

Disability score: 1, normal; 2, walks but cannot run; 3, walks with aids.

Normal range of serum creatine kinase: 25–200 IU/l for male and 25–170 IU/l for female.

### Genetical screening and analysis

All exons and intron-exon junctions of *UBAP1* (GenBank NM_016525.4) were amplified by polymerase chain reaction (PCR). The primers used for screening causal mutations in *UBAP1* are listed in Table 2. Variants in *UBAP1* were confirmed by direct sequencing. In order to excluded other more common genetic causes of HSPs, *SPAST*, *ATL1* and *REEP1* are screened in the individuals with UBAP1 variants. The primers used in the PCR of the three genes are listed in S1–S3 Tables in S1 File, separately. The mutations in Sequencing results were analyzed by Chromas software (Technelysium Pty Ltd). Analysis of amino-acid conservation at and/or near the mutation site was conducted by CLC DNA Workbench.

**Table 2:** Primers used for screening mutation in *UBAP1*.

Exons	Forward (5’→3’)	Reverse (5’→3’)
**Exon 1**	AGAGAGATTAGTTATGTCACCG	TCCGACCCTTCTCCCAGAATC
**Exon 2**	TGGGAGTGAGACGTGAGGATTC	CTATACCTTCAATGCTGCTGAA
**Exon 3**	CTCTGTAACTTCTAGACTTAGG	AACATGATTCAGCTCACAACTG
**Exon 4**	CACCAGGAGTGAGGAAGGAGT	GCAACAGAATGAGACCCTGTC
**Exon 5 & 6**	TAGTGTCAGAAATGCCAACCC	CTTGTACCTGTAGTCACTGGC
**Exon 7**	TTCCACCACAGCCCACCTCT	GGTCCTATTCTATCCCCAGC

### DNA constructs and transfections

The coding region of wild-type UBAP1 (GenBank NM_016525.4) was amplified by PCR from human kidney first-strand cDNA. The wild-type UBAP1 was cloned into a pCMV-Tag2B vector (Agilent Technology) at the sites of *Bam*HI and *Hind* III. The primers used in the UBAP1 construct were as follows: sense 5’-TGACGGATCCATGGCTTCTAAGAAGTTGGG-3’ and antisense 5’-TGACAAGCTTTCAGCTGGCTCCTGCCCGAG-3’. The A157* and L171* mutants were separately constructed by site-directed mutagenesis into the aforementioned vector. All constructs were confirmed by Sanger sequencing.

Neuro-2a cells were obtained from Stem Cell Bank of the Chinese Academy of Sciences (Shanghai, China). The cells were cultured in Dulbecco’s modified eagle medium (DMEM) supplemented with 10% fetal bovine serum (FBS) at 37°C with 5% CO_2_. Cellular transfections were performed with Lipofectamine 3000 (Thermo Fisher Scientific) according to the manufacturer’s protocols.

### Quantitative PCR (qPCR) analyses

Total RNA was extracted and subjected to reverse transcription using the PrimeScript 1st Strand cDNA Synthesis Kit (Takara, Otsu, Japan). Quantitative real-time PCR (qPCR) was performed using the SYBR Premix (Tiangen, Beijing, China). Relative mRNA levels were normalized to that of *GAPDH*. The primers used in qPCR analyses were as follows: UBAP1 (F: 5’-GTTGGGTGCAGATTTTCATGG-3’, R: 5’- CTGTACTTCTCTGACAACCTG-3’); and GAPDH (F: 5’-AGCCACATCGCTCAGACACCA-3’, R: 5’-ATGTAGTTGAGGTCAATGAA-3’).

### Western blotting

For Western blotting, cells were lysed in RIPA (Thermo fisher) with a protease-inhibitor mixture (Roche, Basel, Switzerland). Proteins were separated via sodium-dodecyl-sulfate polyacrylamide gel electrophoresis (SDS-PAGE) under reducing conditions and were then transferred onto polyvinylidene difluoride (PVDF) membranes (0.22 μm, Merck Millipore). After blocking for nonspecific binding, the membranes were probed with antibodies specific to UBAP1 (1:1,000, Abcam, Cambridge, UK) overnight at 4°C, followed by incubation with corresponding secondary antibodies (1:10,000, Thermo fisher) for 1 h at room temperature. Bands were developed on X-ray films (XBT, Kodak, Rochester, NY, USA). GAPDH Protein (primary antibody, 1:5,000, sc-25778, Santa Cruz Biotechnology) was used as the internal control for Western-blotting assays.

### Immunofluorescent analysis

After transfections for 24 h with the indicated plasmids, the localizations of wild-type and mutant UBAP1 in cells were detected. The cells were fixed with 4% paraformaldehyde (Sigma Aldrich) at room temperature for 20 min. After fixation, the cells were washed with phosphate-buffered saline (PBS), and blocked with 5% bovine serum albumin (BSA). The cells were then incubated with anti-FLAG antibody (1:1000, Sigma Aldrich, Shanghai, China) and were then incubated in anti-mouse DyLight® 488 (1:800 dilution; EarthOx, California).

For quantification of neurite outgrowth, neuro-2a cells were transfected with the indicated plasmids and were cultured for 24 h. The neuro-2a cells were treated with RA (10 μM, Sigma Aldrich) and were grown for another 36 h. UBAP1-positive cells were examined by the EVOS FLoid System (Thermo Fisher). We counted the average length of each neurite (i.e., the sum of the length of the primary neurite and the branch lengths). The number of neurites per cell was also quantified. In total, 100 cells per transfection were scored for neurite outgrowth. All samples were performed in triplicate. Statistical analysis was performed by GraphPad Prism, and differences between two experimental groups were assessed by the Student’s t test.

## Results

### Clinical features

In the present study, a total of seven patients from two Chinese families were identified, and the patients presented with consistent or partially related phenotypes. In the first family, the affected proband displayed an abnormal gait with lower-limb hyperreflexia and a Babinski sign. In other patients, limb hypertonia and ankle clonus were also observed. In the second family, both of the two patients I-2 and II-2 presented with HSP signs, including lower-limb hypertonia, hyperreflexia, ankle clonus, and a Babinski sign. Especially, Patient III-2 has either hypertonia or a positive Babinski sign, which reflects the clinical heterogeneity of the disease. In the two families, none of the patients displayed pes cavus, cognitive impairment, ataxia, dysarthria, or any additional abnormal signs. In addition, there were no abnormalities in brain magnetic resonance imaging (MRI), electromyographies, or levels of serum creatine kinase (Table 1). Collectively, our results revealed that the affected individuals displayed lower-limb spastic paralysis without muscle atrophy or brain abnormalities.

### Confirmation of two novel variants in *UBAP1*

**Fig 2:**
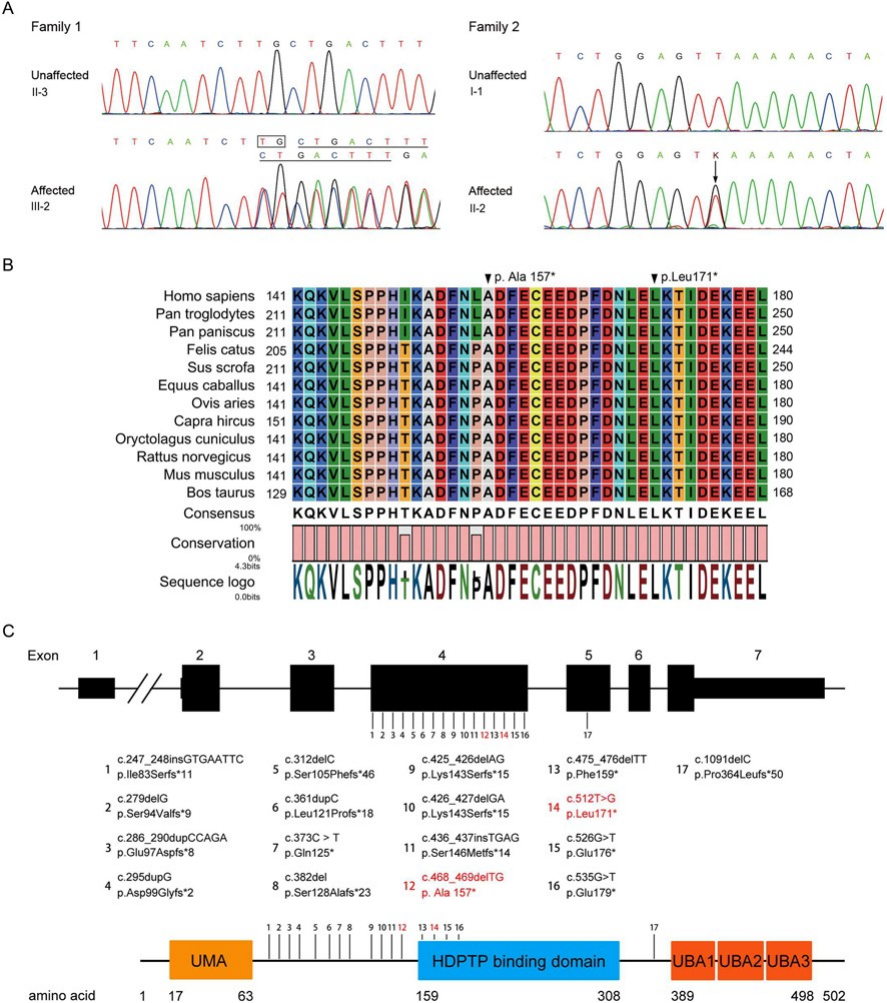
Confirmation of novel variants of *UBAP1*. **(A)** Sequence chromatogram showing the c.468_469delTG (family 1) and c.512T>G (family 2) variants in *UBAP1* in the probands. The variants were numbered according to GenBank NM_016525.4. **(B)** Sequence alignment of mammalian UBAP1 protein showed that the regions around the variants were highly conserved. The numbers on the left and right indicate where the fragment was located within the entire protein sequence. The positions of the variants are marked by black triangles. **(C)** Mutations identified in SPG80. The numbers indicate the locations of mutations in DNA and protein. The mutations found in this study are marked in red.

Mutational screenings were performed for *UBAP1*, from which two heterozygous variants were identified. In the first family, we identified a deletion variant, c.468_469delTG, in all affected individuals. The deletion led to a premature translation termination in *UBAP1* (p.Ala157*, A157*). In the second family, a novel nonsense mutation, c.512T>G, was identified in the two patients (Fig 2A). The nonsense mutation generated a truncated UBAP1, p.Leu171* (L171*). The variants were not detected in unaffected family members or 132 unrelated Chinese controls. Moreover, nonpathogenic variants in *SPAST*, *ATL1* or *REEP1* were detected in the patients. Both mutation sites were located in phylogenetically conserved regions (Fig 2B). These results suggested that the novel mutations may have disrupted UBAP1 function.

**Fig 3:**
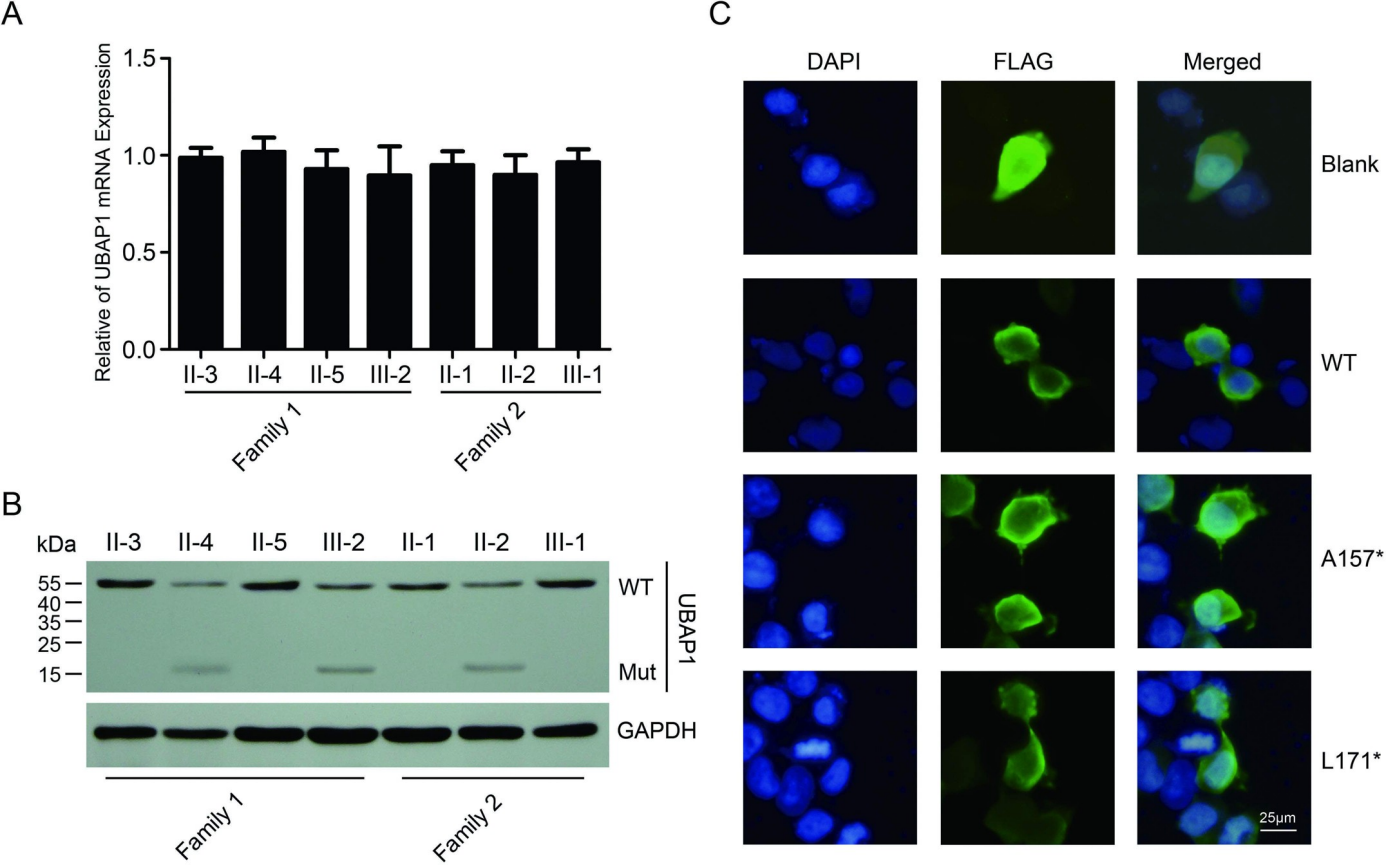
Characterization of A157* and L171* mutants. (A) mRNA expression levels of UBAP1 in lymphocytes were analyzed by qPCR assays. Data were normalized to the levels of GAPDH within each sample (n = 3). The data are presented as the mean ± SE. (B) Protein levels of UBAP1 in lymphocytes were examined by Western blotting. GAPDH was used as an internal control. (C) Localizations of FLAG-tagged A157* and L171* analyzed in neuro-2a cells via immunofluorescent assays showing they did not disrupt the subcellular localization of UBAP1.

At present, there have been seventeen causal mutations in *UBAP1* responsible for HSP, among which, 16 mutations are located in the fourth exon [2, 8–11]. However, this pattern does not indicate that exon 4 is a hot spot for mutations, since this exon occupies most of the coding region of *UBAP1*. All mutants contain the UMA domain, which may represent the binding region with other ESCRT-I subunits (Fig 3C) [8, 14]. We next investigated the functional importance of the novel mutations causing HSP in the present study.

### Characterization *UBAP1* variants

We first analyzed the expression of mutant UBAP1. The two mutations caused the stop codon of *UBAP1* to form prematurely. Mutant transcripts of UBAP1 can be degraded by the nonsense-mediated mRNA decay (NMD) system. In our study, total *UBAP1* transcript levels in patients from the two recruited families were similar to those of unaffected individuals (Fig 3A), which suggested the NMD system did not play a role in the *UBAP1* variants in these two families. To analyze the functions of the variants, we examined the protein expression of UBAP1 in individuals from the two families. Compared to those in unaffected individuals, the mutations in patients resulted in a decreased expression of functional UBAP1 and abnormal production of the truncated protein (Fig 3B). Inaccurate subcellular localization is a potential consequence caused by mutations; hence, we performed immunofluorescent assays to determine this parameter. Both wild-type and mutant UBAP1 were located in cytoplasmic regions of neuro-2a cells (Fig 3C). These results suggest that the novel mutations did not disrupt the subcellular localization of UBAP1.

### A157* and L171* mutants disrupt the neurite outgrowth

To investigate the molecular mechanisms of A157* and L171* mutants in conferring HSP, we examined the effects of the two mutants on neurite outgrowth. The indicated constructs (blank, WT, A157*, and L171*) were transfected into neuro-2a cells and, after treatment with RA, neurite outgrowth was detected by immunofluorescence.

**Fig 4:**
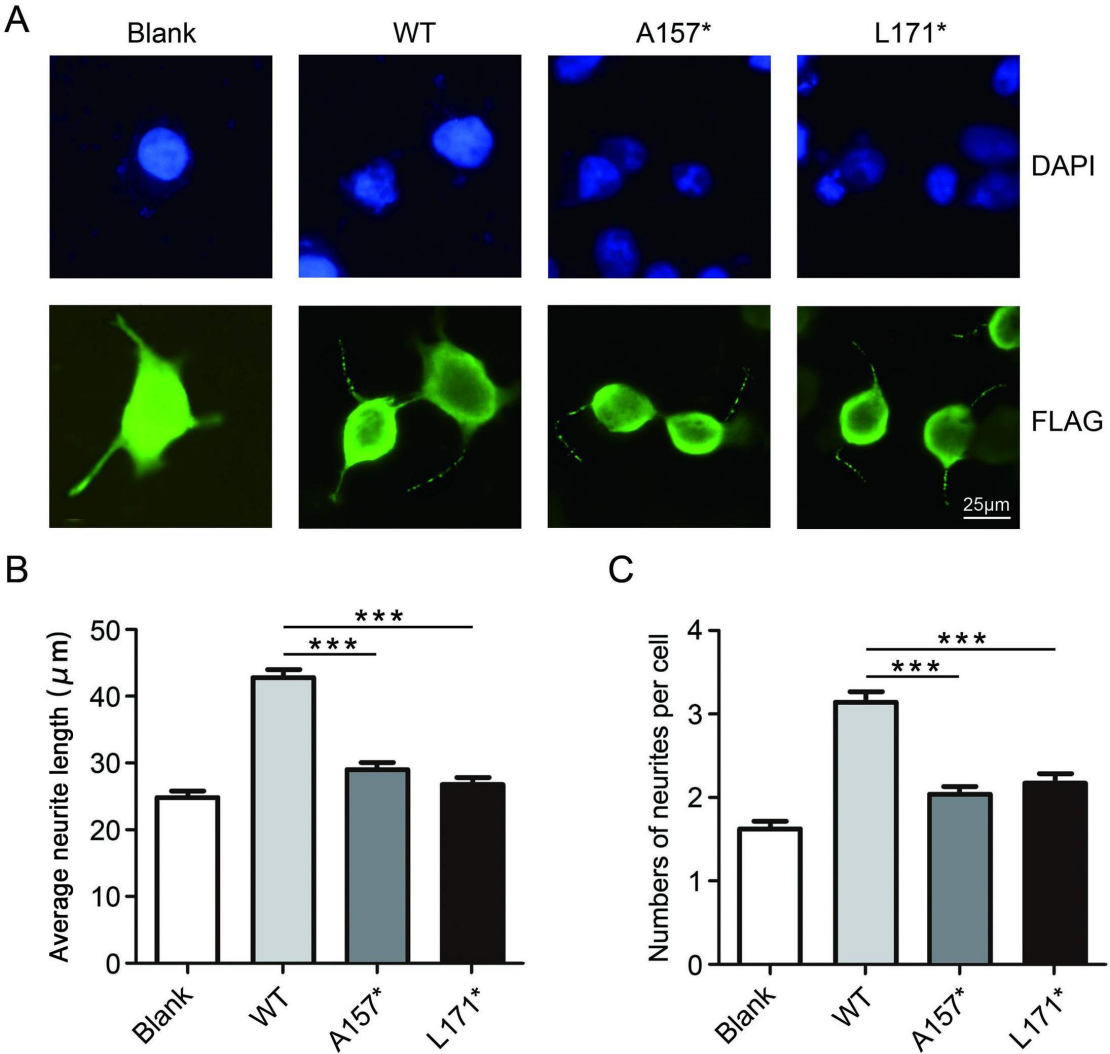
A157* and L171* mutants disrupt neurite outgrowth in neuro-2a cells. **(A)** Neuro-2a cells were transfected with the indicated constructs, and FLAG-positive cells were determined by immunofluorescent assays. **(B) (C)** The average length of each neurite and the numbers of neurites per cell were counted. One hundred neurites in FLAG-positive cells were scored for neurite outgrowth. Neurite numbers were counted in one hundred FLAG-positive cells. The data are presented as the mean ± SE (***p < 0.0001).

Neurite outgrowth of neuro-2a cells was induced by RA (Figs 3C and 4A). Overexpression of wild-type UBAP1 increased the lengths and numbers of neurites. Compared to that of wild-type UBAP1, A157* and L171* mutants reduced the neurite outgrowth by 31.7% and 33.6%, respectively. The number of neurites decreased from approximate three to two (Fig 4B and 4C). Our results suggest that A157* and L171* mutants lost the function to enhance the formation and extension of neurites.

## Discussion

We identified two novel mutations of *UBAP1* in two separate Chinese families with HSP. In the first family, a deletion variant in *UBAP1*, c.468_469delTG, was responsible for HSP. In the second family, a nonsense mutation, c.512T>G, caused HSP. Both of these mutations led to the formation of truncated UBAP1. Moreover, UBAP1 mutants disrupted neurite outgrowth in neuro-2a cells.

*UBAP1* is originally derived from the tumor-suppressor locus in human chromosome 9p21–22 [15]. Most recently, mutations in *UBAP1* were identified to be responsible for a subset of HSP [2]. In less than two years, the pathogenicities of *UBAP1* mutations in HSP have been reported in multiple studies [8–11]. Interestingly, all of these mutations cause UBAP1 to form truncated proteins. However, none of the mutations impair the UMA domain, and mutant UBAP1 can still form ESCRT-I complexes via its other three subunits (Fig 2C) [8, 14].

In the present study, the patients in the two families had juvenile-onset HSP with autosomal dominant inheritance. Clinically, this is a pure form of HSP with a slow progression. However, it is not phenotypically distinct from other pure HSPs. Also, on the early stage of HSP, the patients may have no typical signs of HSP (Table 1), which adds to the difficulty of diagnosis. Although two novel mutations of *UBAP1* were identified, no *UBAP1* mutations were found in other patients recruited with similar phenotypes. These findings demonstrate that HSPs are complex and are both clinically and genetically heterogeneous.

UBAP1 is a multi-domain protein. The N-terminal consists of an UBAP1-MVB12-associated (UMA) domain (AA 17–63) responsible for binding to other ESCRT-I subunits [13]. The SOUBA domain, in the C-terminal, is responsible for interactions with ubiquitin (Fig 2C) [14]. UBAP1 binds to the PTPN23 protein through its HDPTP-binding domain (AA 159–308), which functions in endosomal cargo sorting and multivesicular-body morphogenesis [16, 17].

UBAP1 is a subunit of the ESCRT-I [14], and loss of function of this complex is a possible cause of HSP. It has been reported that mutations of *UBAP1* impair endosomal processing, which is an important function of ESCRT-I [8]. This finding is suggestive of the functional importance of ESCRT-I in HSPs, but does not explain why there are no other types of causal mutations, such as missense mutations, responsible for HSPs. Moreover, UBAP1 is ubiquitously expressed and is highly expressed in the heart, brain, placenta, lung, liver, skeletal muscle, and pancreas [15]. However, mutations in UBAP1 cause pure HSP rather than a complicated form. Currently, little research has been conducted on the molecular function of UBAP1. In sporadic studies, UBAP1 plays a role in proteasomal degradation of ubiquitinated cell-surface proteins [8, 13, 14, 18]. The intrinsic function of UBAP1 alone, rather than within the ESCRT-I complex, also needs to be further investigated. Mutations in VPS37A, another subunit of ESCRT-I, can also cause HSP [19]. Intriguingly, the HSP caused by *UBAP1* mutations are autosomal dominant, while mutations of VPS37A cause autosomal-recessive HSP. This suggests that the two proteins have different expression profiles, and that each of the subunits may exert differential roles.

KIDINS220 variants can cause complex spastic paraplegia and have been found to have no effect on neurites in differentiated neuro-2a cells [20, 21]. However, in our present study, we found that truncated UBAP1 impaired neurite outgrowth (Fig 4). It is not clear whether UBAP1 acts as a monomer or a unit of ESCRT-I in differentiated neuro-2a cells. Importantly, HSPs are characterized by progressive degeneration of the corticospinal tract [22]. Other studies have already highlighted the importance of Spastin which is a well-established gene causing HSP in the neurite outgrowth [23]. Nevertheless, whether neurites play a role in the pathophysiology of HSPs requires further investigation.

In our study, we took an atypical approach to identify the causative gene of HSP. We examined *UBAP1*, the newly reported pathogenic genes of HSP [2], in our families and identified two novel variants. We believe that the two novel variants are responsible for HSP from a medical genetics point of view. In fact, in routine screening for disease-causing genes, the most common autosomal dominant HSPs, such as SPG4, SPG3, and SPG31, should be detected first. This, however, does not seem to be the best way to explore the pathogenic genes of HSPs. We have examined the common candidate genes (including *UBAP1*) of pure HSPs in the remaining samples, but no pathogenic mutations were found (data not shown). The clinical phenotypes of several patients are highly similar to those in the present study. Considering the genetic heterogeneity of HSPs, next-generation sequencing is the preferred method for studying HSP pathogenic genes.

In summary, we identified two novel variants in *UBAP1* in two Chinese families as the probable cause of HSP. The mutations resulted in truncated proteins of UBAP1 that impaired neurite outgrowth. Taken together, our findings expand the pathogenic spectrum of *UBAP1* variants in HSP.

## Supporting information

S1 Raw image (PDF)

S1 Raw data Raw data of Figs 3A, 4B and 4C. (PDF)

S1 File (PDF)
