# Supplementary figures and images for: Two novel truncating variants in UBAP1 are responsible for hereditary spastic paraplegia
Source: PLoS One. 2021 Jun 30;16(6):e0253871. doi: 10.1371/journal.pone.0253871 (PMC8244911; doi:10.1371/journal.pone.0253871)

S1 Raw image

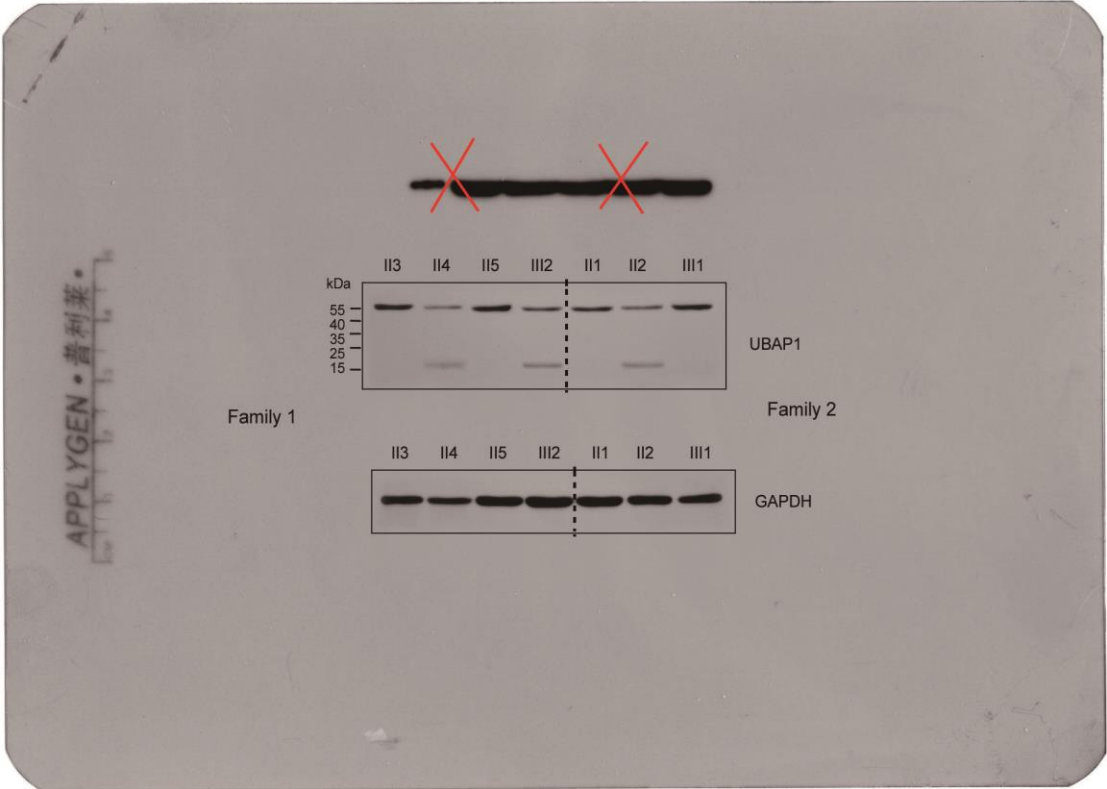

Supplement: S1 Raw image — (PDF) [file pone.0253871.s001.pdf]
