# Supplementary material for: Two novel truncating variants in UBAP1 are responsible for hereditary spastic paraplegia
Source: PLoS One. 2021 Jun 30;16(6):e0253871. doi: 10.1371/journal.pone.0253871 (PMC8244911; doi:10.1371/journal.pone.0253871)
Supplement: S1 Raw data — (PDF) [file pone.0253871.s002.pdf]

S1 Raw data Relative Expression of UBAP1 mRNA*

Data family 1 ($\times 10^{-4}$)				Data family 2 ($\times 10^{-4}$)		
**II3**	**II4**	**II5**	**III2**	**II1**	**II2**	**III1**
4.992840	5.008471	5.002567	6.338324	5.009056	5.907181	4.892294
5.547238	6.221952	4.305387	3.767323	5.847007	4.564930	5.085407
5.708424	5.510084	5.985883	4.586735	4.777011	4.348765	5.866733

* The data was normalized to GAPDH.

Table 2 The neurite length of neuro-2a*

**Blank**	**WT**	**A157***	**L171***
17	57	29	23
23	33	24	27
14	43	25	25
18	64	37	37
27	41	29	28
26	59	19	22
33	27	16	35
33	34	27	19
30	43	26	28
25	52	24	22
29	31	43	29
19	44	15	13
23	61	26	35
21	38	34	27
29	36	23	27
22	40	35	45
27	42	17	15
21	34	34	31
23	44	33	29
22	50	35	10
23	21	49	8
24	35	24	28
21	60	42	30
30	43	31	25
19	40	22	28
17	53	36	26
21	69	31	34
30	29	34	24
22	38	37	36
20	56	33	30
11	28	19	10
32	52	30	34
25	46	46	15
27	49	19	26
28	33	25	26
33	50	29	31
16	27	19	30
11	25	29	32

30	59	33	26
22	51	22	31
19	41	34	24
20	41	25	37
26	59	29	33
29	52	29	31
33	64	33	23
31	49	19	27
32	55	24	17
29	45	25	11
34	38	27	22
16	52	25	26
22	36	22	40
25	28	33	37
31	41	28	35
29	45	17	11
20	36	15	16
29	51	31	29
25	20	21	23
30	46	19	20
41	17	22	33
24	33	37	33
29	39	41	25
11	43	36	34
19	43	30	28
28	60	20	28
37	42	26	23
16	35	27	34
32	37	46	24
22	42	22	13
38	39	35	17
29	44	25	31
14	40	27	30
27	52	42	19
24	63	28	20
19	50	16	25
23	45	32	34
34	38	24	34
28	37	30	35
34	44	12	25
31	38	36	33
21	42	29	38
26	50	23	24

34	59	42	32
35	36	37	25
26	35	21	29
38	47	37	21
27	38	35	24
14	36	36	24
25	50	34	29
31	52	27	38
28	31	31	26
23	19	23	24
18	23	31	34
22	34	46	33
11	33	31	34
21	58	39	19
21	55	35	31
20	58	38	18
20	35	17	19
24	39	22	35
18	34	39	34

* The units of value are μm.

Table 3 The number of neurites in neuro-2a

Blank	WT	A157*	L171*
0	6	2	4
1	5	1	1
2	3	1	1
2	1	4	3
1	1	3	2
2	3	1	1
1	4	3	0
1	3	2	3
2	3	2	1
2	3	3	3
3	3	2	2
3	3	2	2
1	2	2	1
2	5	3	4
1	3	2	1
1	3	3	2
3	2	2	4
2	3	2	3
3	2	2	2
4	1	2	2
1	2	2	2
2	6	1	3
2	2	2	2
2	3	2	2
1	4	2	3
1	4	3	3
2	4	1	2
1	5	2	1
3	4	1	2
1	6	2	3
2	4	2	3
2	1	3	2
2	3	0	3
2	4	3	3
0	3	1	3
2	3	3	2
0	3	2	3
1	4	3	2

3	3	2	3
2	2	2	2
2	2	1	1
1	4	3	1
1	3	1	0
3	4	3	2
0	2	2	3
3	4	2	-1
3	1	2	2
1	1	3	1
1	3	2	0
1	4	1	1
2	4	2	4
1	5	2	3
1	4	1	3
1	2	1	3
3	4	1	3
2	3	3	2
2	2	3	4
1	2	2	1
1	5	1	3
1	2	1	2
1	2	2	4
2	4	2	2
1	1	3	2
2	3	1	4
1	2	2	3
2	1	1	2
2	3	3	1
1	3	3	2
1	5	2	1
3	2	3	2
2	4	4	3
2	2	1	2
2	1	1	2
2	3	2	1
1	0	3	2
2	4	3	1
1	3	2	2
1	3	1	0
2	1	3	2
2	5	2	2
2	3	2	3

1	3	2	1
3	4	2	1
2	5	2	4
1	3	2	0
0	3	2	3
2	4	2	2
1	4	2	1
1	5	4	3
1	5	3	2
2	3	2	3
2	4	2	3
2	4	1	4
2	3	1	3
0	2	2	3
0	1	2	2
1	4	1	2
2	4	2	3
2	3	2	3
2	4	2	2
