# Supplementary material for: Two novel truncating variants in UBAP1 are responsible for hereditary spastic paraplegia
Source: PLoS One. 2021 Jun 30;16(6):e0253871. doi: 10.1371/journal.pone.0253871 (PMC8244911; doi:10.1371/journal.pone.0253871)
Supplement: S1 File — (PDF) [file pone.0253871.s003.pdf]

S1 File Primers used for screening mutation in *SPAST*

Exons	Forward (5’→3’)	Reverse (5’→3’)
**Exon 1**	GACGTGAGCCGAACTGCACAT	TCCGACCTACGGGAAAGCAGT
**Exon 2**	ATGTATTACCTCTCAACAGCA	GATCTGAAATCTGGACAATCA
**Exon 3**	TGATTCTGCTTTGTCTGCCCA	GGACCACATTTTCAATCACTG
**Exon 4**	AGTAATTTGTCATTTCACATGC	ACTATATTTTCAACACAGCCC
**Exon 5**	TGTTTGCTTGTCTTTATGTTCA	GGAAACTTATCCATTCTCTAA
**Exon 6&7**	AGCTTGAATTCTGTGAACTTTAA	CTACTACTACTATGGATTCAG
**Exon 8**	AGCTATGGGCAGCTCTGTTTG	CGTAAATAATAGACTCAAGGAC
**Exon 9**	CCTGGCCTCATAGCTTACATT	TTTAAGCCAGCCAGTTTACGG
**Exon 10&11&12**	ATTCCTGTGTGCTAGATTTTC	GACCTCAAAATCCTTATGTAG
**Exon 13**	CTAGGCAGTTCACCTTTATGT	GAAATTGCTGGCTCTGACAGT
**Exon 14**	GTGCCTTTGGTCCCAGTTACT	GCAAAGGAGGTAGAGGATGAG
**Exon 15**	GCCTGACCAACATGGCGAAAC	GCAACAGAGTGAGACCCTGTC
**Exon 16**	TGTACTTGGTTTTGCCCTTCA	AGGAGGTGGAGGTTGCAGTGA
**Exon 17-1**	GCAGCATCATTACTTTAATCC	CATGATATGAGTCTCTACTCC
**Exon 17-2**	TGTACAGTAATTCCATTTGTTTG	AGAGCCACCCAATCATTGCTT

Table S2 Primers used for screening mutation in *ATL1*

Exons	Forward (5'→3')	Reverse (5'→3')
**Exon 1**	CGTAGCCCTCCAAGTTAAACA	CCTGTTCCATATATGCTGTGC
**Exon 2**	GTGGTCTATCACTGTCTTCAC	GTGGACGCAGGCACCTAGCA
**Exon 3**	CGGATGTTTGAGAGTTAAGAG	TTGGAATGGTTACACCACAGC
**Exon 4&5**	GGTATCAATGCAATAGATGTG	ATGACCTTTTCTAACCAAAGC
**Exon 6**	CTACTGTATTTCTGGAACCAT	TCGGCCATGACTGTAAATTTC
**Exon 7**	ATTCTGTTATACCTAGAGGGA	TCATCTTTCATTTCCACATCT
**Exon 8**	TGTAGGATGATCTCATAGGGG	TGATATTTGCACTGTCAGTCT
**Exon 9**	CGTGTCATTTTCATCATTGTG	AGAAGGTGAGGGTTACTGCCT
**Exon 10**	GTTTCAGGAGTCGCTTAAATG	TCAAAATACAGCTCCCTCAAG
**Exon 11**	TGGCAGACAGAGATGTTTGAA	AACTCCTGGGCTCAAGTGATC
**Exon 12**	TGGTTTCTTGCACATTTCTTG	CGAGGGTACCACATGCTATTT
**Exon 13**	CAGTCCCATTTATAGTCATGC	ACTGGAAAATAGTTGTGTAGG
**Exon 14**	CAACATGCTAAATTTTATACAGTA	TTAAGTTTGTACTGATGTCAACA

Table S3 Primers used for screening mutation in *REEP1*

Exons	Forward (5’→3’)	Reverse (5’→3’)
Exon 1	TGAGGCTAACGCACTAGCTGAA	CAAATCGACCGAGCTGAGGAA
Exon 2	AGCACTTTCTGTAGCTTAGAG	TCCAGTGCCCATAGCACGGA
Exon 3	GATAGGGAGAAGGCTACAAAT	TGCTCCAGTTAACATCCCTGC
Exon 4	CAGCCACATGCAAGCAGGAGA	ATGAGAGCCAAATGAGAAGTC
Exon 5	CCAGCAAGAACAAGGATTGGG	TGGTCCTTAGCCTGTTCTGTG
Exon 6	TGCGCCTCAGAGTTGATCCAG	CCTGGCATGATCTGATGGACA
Exon 7-1	GGAGCTGGAAGTCTCAACACA	TCGGACCACAATGCCATTTAA
Exon 7-2	GCTTAGTAGGGGAATAAGGCA	CCATTTCAAATACTGTGCCAG
Exon 7-3	CCATGTGTTTGTGATAAGAGT	TTCACAGCTACCTAGTTTCTG
